# Comparison of four tools to identify painful new osteoporotic vertebral fractures in the postmenopausal population in Beijing

**DOI:** 10.3389/fendo.2022.1013755

**Published:** 2022-11-08

**Authors:** SiJia Guo, Ning An, JiSheng Lin, ZiHan Fan, Hai Meng, Yong Yang, Qi Fei

**Affiliations:** Department of Orthopedics, Beijing Friendship Hospital, Capital Medical University, Beijing, China

**Keywords:** osteoporosis, postmenopausal, vertebral fracture, FRAX, BMD, BFH-OST, OSTA

## Abstract

**Objectives:**

To validate and compare four tools, the Fracture Risk Assessment Tool (FRAX) without bone mineral density (BMD), Beijing Friendship Hospital Osteoporosis Screening Tool (BFH-OST), Osteoporosis Self-Assessment Tool for Asians (OSTA), and BMD, to identify painful new osteoporotic vertebral fractures (PNOVFs).

**Methods:**

A total of 2874 postmenopausal women treated from June 2013 to June 2022 were enrolled and divided into two groups: patients with PNOVFs who underwent percutaneous vertebroplasty (PNOVFs group, n = 644) and community-enrolled females (control group, n = 2230). Magnetic resonance and X-ray imaging were used to confirm the presence of PNOVFs. Dual-energy X-ray absorptiometry was performed to calculate the BMD T-scores. Osteoporosis was diagnosed according to WHO Health Organization criteria. Data on the clinical and demographic risk factors were self-reported using a questionnaire. The ability to identify PNOVFs using FRAX, BFH-OST, OSTA, and BMD scores was evaluated using receiver operating characteristic (ROC) curves. For this evaluation, we calculated the areas under the ROC curves (AUCs), sensitivity, specificity, and optimal cut-off points.

**Results:**

There were significant differences in FRAX (without BMD), BFH-OST, OSTA, and BMD T-scores (total hip, femoral neck, and lumbar spine) between the PNOVFs and control groups. Compared with BFH-OST, OSTA, and BMD, the FRAX score had the best identifying value for PNOVFs; the AUC of the FRAX score (optimal cutoff =3.6%) was 0.825, while the sensitivity and specificity were 82.92% and 67.09%, respectively.

**Conclusion:**

FRAX may be the preferable tool for identifying PNOVFs in postmenopausal women, while BFH-OST and OSTA can be applied as more simple screening tools for PNOVFs.

## Introduction

Primary osteoporosis is a systemic metabolic disease characterized by bone mass loss, impaired bone microarchitecture, and increased bone fragility (1). Postmenopausal osteoporosis (type I) is one of the most common primary forms of bone loss encountered in clinical practice^2^. The clinical outcome of osteoporosis is fragility fractures, of which vertebral fractures are the most common. The prevalence of vertebral fractures in women over 50 years old in China is 15%, while it can reach as high as 36.6% among women aged 80 years or older (2). An initial vertebral fracture is generally accepted as a major risk factor for new fractures (3). A previous study reported that the presence of one or more vertebral fractures increased the risk of sustaining a vertebral fracture by 5-fold in the first year, and that 20% of affected women will experience another fracture within the first year of a vertebral fracture (4). The annual cost of vertebral fractures among women in the United States was $663 million in 2005, and this cost is expected to increase by more than 53% by 2025 (5).

Early identification of painful new osteoporotic vertebral fractures (PNOVFs) is still challenging worldwide, especially in communities and primary medical institutions. The clinical onset of PNOVFs is often hidden, as affected patients generally only have a history of mild low-energy injuries, or even no trauma history at all. Furthermore, the degree of pain varies greatly, with some patients developing chronic pain, while physical examination often does not reveal any clear localization signs, and it should be noted that some patients complain that the pain site is not consistent with the actual fracture level (6). These factors may all contribute to mis- and missed diagnosis, especially in communities and primary medical institutions with limited professional experience and equipment. As such, there is an urgent need to identify a reliable, simple, and cost-effective tool for screening PNOVFs in postmenopausal women.

Bone mineral density (BMD) is the gold standard for diagnosing osteoporosis using dual-energy X-ray absorptiometry (DXA). Osteoporosis can be diagnosed when an individual’s T value for BMD is 2.5 standard deviations or more below the average of young adult women (7). Previous studies have indicated that bone mineral density (BMD) is the best predictor of fractures in perimenopausal women (8). However, BMD only accounts for 60-70% of the variation in bone strength, and therefore does not provide a complete picture of bone strength (9). It has been reported that approximately 36.21% to 55.91% of patients with fragility fractures in the postmenopausal population have T-scores above the osteoporotic threshold (10). However, the high cost of DXA machines prevents their widespread use in primary hospitals, particularly in developing countries. Moreover, DXA examinations involve exposure to ionizing radiation, making this procedure highly complex, expensive, and inconvenient. As a result, a convenient and economical tool for PNOVFs screening is urgently needed.

The FRAX (https://www.sheffield.ac.uk/FRAX) is a computer-based tool used to assess the probability of a 10-year hip fracture or major osteoporotic fracture in male and female patients. Several studies have validated the FRAX for identifying PNOVFs in China, but the optimal threshold varies greatly among previous studies (11, 12). Therefore, the use of FRAX in China should be reconsidered. In addition, it has been reported that the use of FRAX without BMD had approximately the same performance as BMD without FRAX (13). As such, it is necessary to validate the FRAX and to determine the optimal threshold for identifying PNOVFs.

The OSTA is a screening tool developed and validated in eight Asian countries to screen for postmenopausal osteoporosis in Asian populations. The OSTA index can be used to identify women at low (index > -1), intermediate (index –1 to -4), and high (index < –4) risk of osteoporosis (14). Our previous study showed that OSTA was a valuable tool for identifying PVNOFs in a population of 1201 postmenopausal women (15). However, it is still unknown whether this is the best tool to identify PNOVFs.

The Beijing Friendship Hospital Osteoporosis Screening Tool (BFH-OST tool) was developed based on community-dwelling postmenopausal Han Chinese women in Beijing, and includes four clinical risk factors: history of fragility fracture, age, height, and weight (16). Previous studies have confirmed that this tool can accurately identify postmenopausal osteoporosis, with a sensitivity of 73.6% and a specificity of 72.7% for identifying osteoporosis at a cutoff of 9.1 according to the WHO criteria, with an area under the receiver operating characteristic curve (AUC) of 0.797 (16). However, it is unclear whether this tool has any value in detecting and identifying PNOVFs.

This study aimed to compare and validate OSTA, BMD, FRAX, and BFH-OST, to identify PNOVFs and determine the optimal threshold.

## Patients and methods

**Figure 1:**
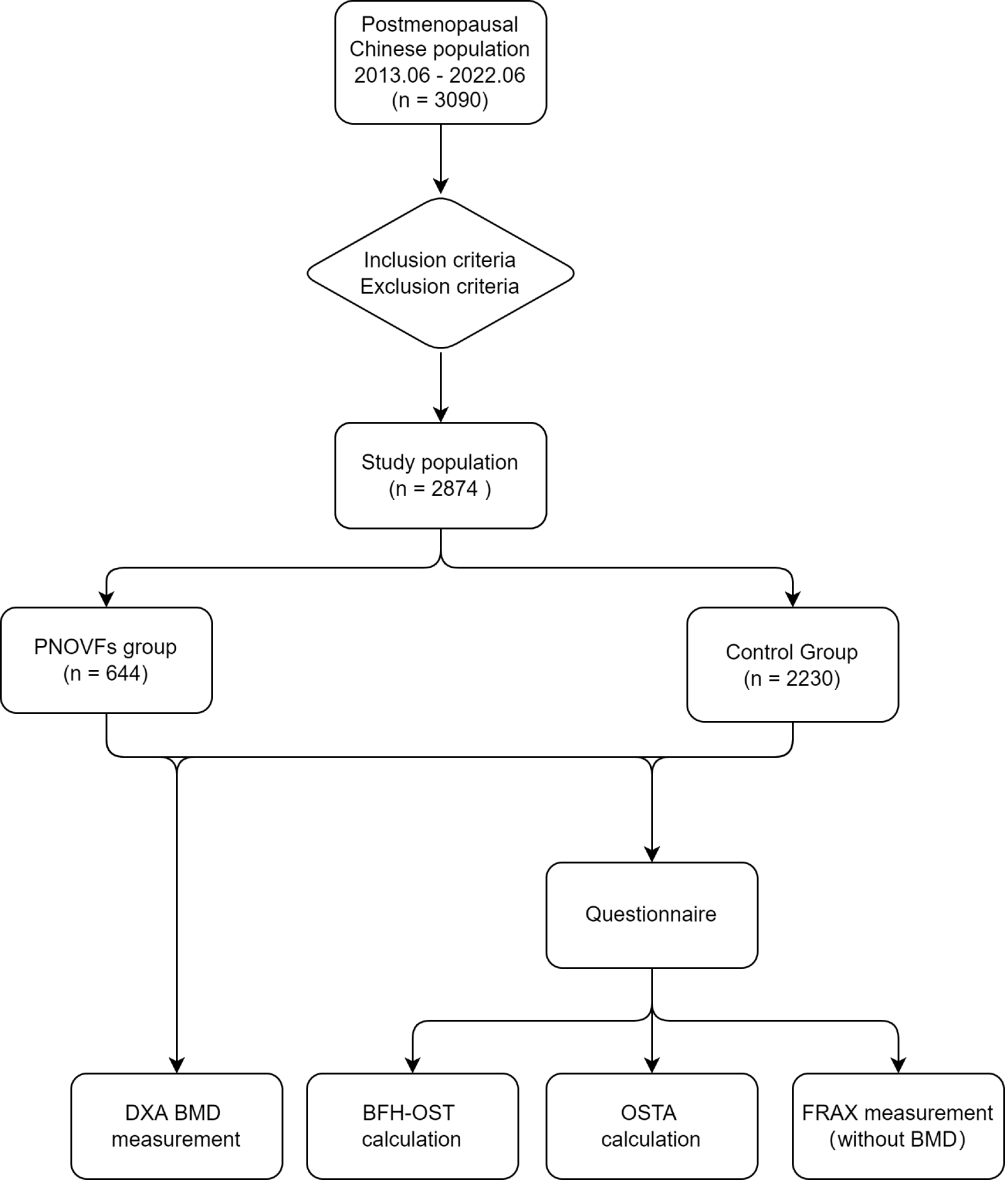
Flowchart of the study.

This retrospective study was approved by the Ethics Committee of Beijing Friendship Hospital, Capital Medical University. All participants provided written informed consent to participate in the study. A flowchart of the study is shown in **Figure 1**.

### Study design

The study population included postmenopausal Chinese women consecutively recruited from the Osteoporosis Clinic of Beijing Friendship Hospital from June 2013 to June 2022. The cohort comprised clinically symptomatic patients with PNOVFs verified by X-ray and MRI within the past 6 months who presented for further examination and treatment (PNOVFs group), as well as community-enrolled women who presented to our hospital for routine health examinations (control group). The inclusion and exclusion criteria are listed in **Table 1**.

**Table 1:** Inclusion and exclusion criteria for this study.

Inclusion criteria	Exclusion criteria
Han Chinese nationality	A history or evidence of metabolic bone disease
Postmenopausal women	History of organ transplant;
Residing in Beijing ≥ 20 years	Prior use of anti-resorptive or anabolic agents
	Cancer with metastasis to the bone
	Significant renal impairment
	A condition of prolonged immobility

### BMD measurements and identification of PNOVFs

All participants underwent DXA BMD measurement (Hologic Inc., Bedford, MA, USA) of the hip and spine, and were interviewed by a trained interviewer using a standardized questionnaire investigating participants’ demographic and clinical risk factors. To standardize measurements, all DXA scans were conducted by the same technician who was well-trained and qualified. The DXA machine was calibrated by the same technician every day by using a lumbar module. There were less than 50 cases of BMD measurements per day to ensure the accuracy of the results. The following data were collected: age, weight, height, previous fracture, parent-fractured hip, current smoking, glucocorticoid use, history of rheumatoid arthritis, and alcohol consumption per day. The database was developed by two researchers (Sijia Guo and Ning An) in order to guarantee the accuracy of the data, and on the second day, another senior researcher (Yong Yang) verified it. The corresponding author completed the final data entry in order to confirm that the analysis and confirmation of the data were done objectively. If a mistake was made, it would be corrected by going back to the patient’s answers on the questionnaire. According to the WHO criteria, osteoporosis is defined as a T-score (lumbar spine, femoral neck, or total hip) −2.5 standard deviations or lower than that of the average young adult.

Following identification of PNOVFs, data for the following four previously reported clinical criteria were collected: (1) postmenopausal status without trauma history or with a low-energy trauma history (low-energy trauma fracture was defined as a fracture resulting from a fall from a standing position or lower); (2) pain occurring within 6 months prior to BMD measurement; (3) acute or subacute vertebral fractures with correlating clinical signs and signs demonstrated by X-ray (i.e., height loss in the anterior, middle, or posterior dimension of a vertebral body that exceeds 20% of the vertebral body area in a lateral-view image of the thoracic/lumbar spine; or the presence of endplate deformities, a lack of parallelism of the endplates, and a generally altered appearance relative to neighboring vertebrae) and spine MRI imaging (new bone marrow edema apparent in sagittal T1-weighted and fat-suppressed T2-weighted images); and (4) no history or indicative evidence of metabolic bone disease or cancer (15).

### FRAX score

The FRAX is a computer-based algorithm used to calculate the 10-year probability of major osteoporotic and hip fractures. FRAX scores were calculated based on clinical risk factors, for which optional BMD could enhance their prediction efficacy. The FRAX models are available in China. To identify PNOVFs in this study, FRAX (without BMD) scores for the 10-year probability of major osteoporotic fractures were obtained.

### BFH-OST

The BFH-OST was calculated from the following formula (16):

BFH-OST = [body weight (kg) – age (years)] ×0.5+0.1× height (cm) -[previous fracture (0/1)]

For example, a 70-year-old woman with a body weight of 50 kg, height of 160 cm, and a previous fracture would have a BFH-OST index of 5.

### OSTA

The OSTA was calculated based on age and body weight using the following formula (14):

OSTA = [body weight (kg) - age(years)] × 0.2

The decimal points of the calculation results were disregarded. For example, a 71-year-old woman with a body weight of 50 kg would have an OSTA score of -4.

### Statistical analysis

Categorical variables are grouped and presented as numerical values, and continuous data are presented as mean ± standard deviation. The Kolmogorov-Smirnov test was used to test the data distribution. Normally distributed data were assessed using the t-test, whereas the Mann-Whitney U test was applied for non-normally distributed data. Categorical data were analyzed using the chi-squared test. The diagnostic value was assessed using the receiver operating characteristic curve (ROC), and the area under the curve (AUC) and 95% confidence interval (CI) were subsequently calculated. The predictive efficacies of the above tools were estimated according to the AUC values as follows: AUC=1, perfectly predictive; 0.9≤AUC <1, highly predictive; 0.7≤AUC<0.9, moderately predictive; 0.5≤AUC<0.7, less predictive; and AUC<0.5, non-predictive (17). Statistical significance was set at P < 0.05. All data analyses were performed using SPSS v25.0 software (IBM Corp., Armonk, NY, USA), and graphics were drawn using OriginPro 2022 (OriginLab, Northampton, MA, USA).

## Results

A sample of 3090 women were initially included in this study. In total, 216 subjects were excluded from the study for meeting the exclusion criteria, so 2874 subjects were analyzed. This cohort included 644 women with PNOVFs within 6 months before the BMD measurement (PNOVFs group), as well as 2230 community-enrolled women (control group) without specific osteoporosis-associated symptoms. The demographic characteristics of the study participants are shown in **Table 2**.

**Table 2:** Summary of descriptive characteristics of PNOVFs Group and Control Group.

Characteristics	PNOVFs group	Control group	p (t/χ2)
Subjects, n	644	2230	
Weight, kg	58.44 ± 10.42	61.58 ± 9.24	<0.001 (4.141)
Age, year	72.76 ± 8.46	61.11 ± 8.57	<0.001 (-30.488)
Height, cm	157.23 ± 5.36	158.94 ± 5.13	<0.001 (4.359)
BMI, kg/m^2^	23.61 ± 3.93	24.37 ± 3.45	<0.001 (2.664)
Previous fracture	150 (23.3%)	318 (14.3%)	<0.001 (29.901)
BMD, g/cm^2^			
Femoral neck	0.570 ± 0.125	0.700 ± 0.128	<0.001 (9.052)
Total hip	0.678 ± 0.139	0.802 ± 0.136	<0.001 (9.998)
Lumbar spine	0.731 ± 0.146	0.869 ± 0.159	<0.001 (8.118)
Family history, n	70 (10.9%)	270 (12.1%)	0.391 (0.734)
Current smoker, n	39 (6.1%)	55 (2.5%)	<0.001 (20.351)
Alcohol > 30 g/d, n	11 (1.7%)	32 (1.4%)	0.615 (0.253)
Rheumatoid arthritis, n	16 (2.5%)	107 (4.8%)	0.011 (6.350)
Glucocorticoids taking, n	15 (2.3%)	90 (4.0%)	0.042 (4.135)

Data are presented as n (%) or mean ± standard deviation.

BMD, bone mineral density.

Age, weight, height, and BMI were all lower in the PNOVFs group than in the control group (**Table 2**, P<0.001). The PNOVFs group had a greater proportion of previous fractures, current smokers, rheumatoid arthritis, and history of glucocorticoid use. Moreover, the PNOVFs group had lower average BMD values and T-scores at the total hip, femoral neck, and lumbar spine than in the control group (**Table 3**, P<0.001). A higher FRAX value (without BMD) was observed in the PNOVFs group (**Table 3**, P<0.001).

**Table 3:** BMD T-score, BFH-OST, and FRAX scores of the PNOVFs group and control group.

Parameter	PNOVFs group	Control group	z/t	P-value
Subjects, n	644	2230		
BMD T-score				
Total hip	-1.996 ± 1.227	-0.678 ± 1.213	24.209	<0.001
Femoral neck	-2.463 ± 1.191	-1.396 ±1.049	22.038	<0.001
L1-L4	-2.669 ± 1.325	-1.085 ± 1.394	25.678	<0.001
BFH-OST	8.331 ± 7.529	15.983 ± 6.534	25.268	<0.001
OSTA	-2.600 ± 2.720	0.060 ± 2.211	22.8	<0.001
FRAX (%)	6.606 ± 3.278	3.460 ± 2.189	-28.416	<0.001

BMD, bone mineral density; BFH-OST, Beijing Friendship Hospital Osteoporosis Screening Tool; OSTA, Osteoporosis Self-Assessment Tool for Asians; FRAX, Fracture Risk Assessment Tool.

**Figure 2:**
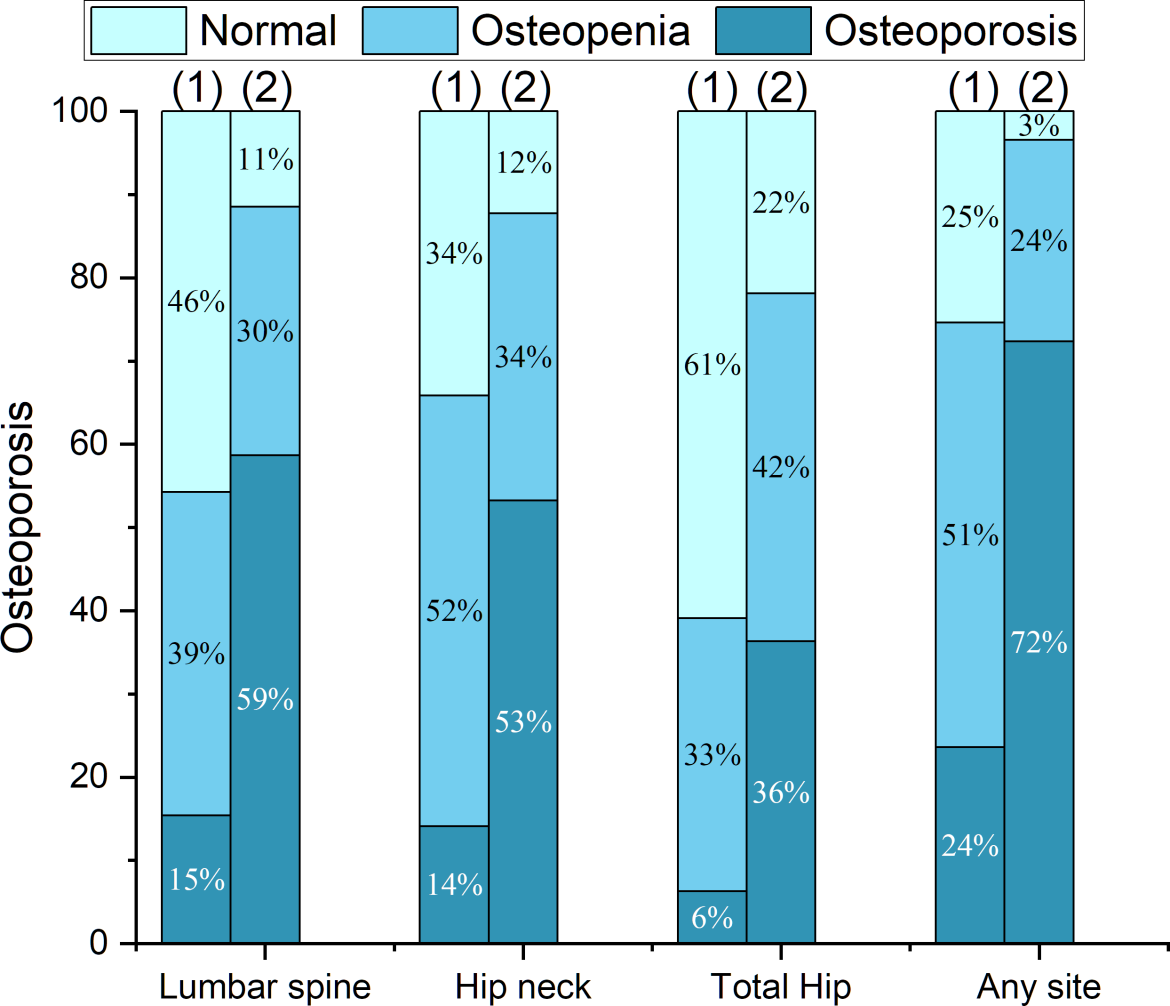
Proportions of BMD T-scores at different sites in the fracture and control groups, including: (1) the control group; (2) the PNOVFs group.

**Figure 3:**
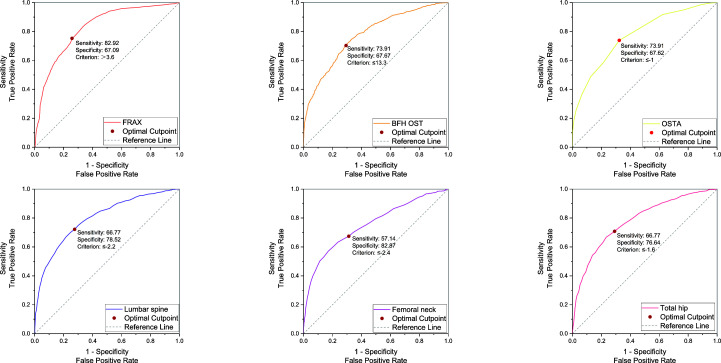
ROC curve of the FRAX without BMD, BFH-OST, OSTA, and BMD T-score (femoral neck, total hip, and lumbar spine) for identifying PNOVFs with optimal cutoff value.

**Figure 4:**
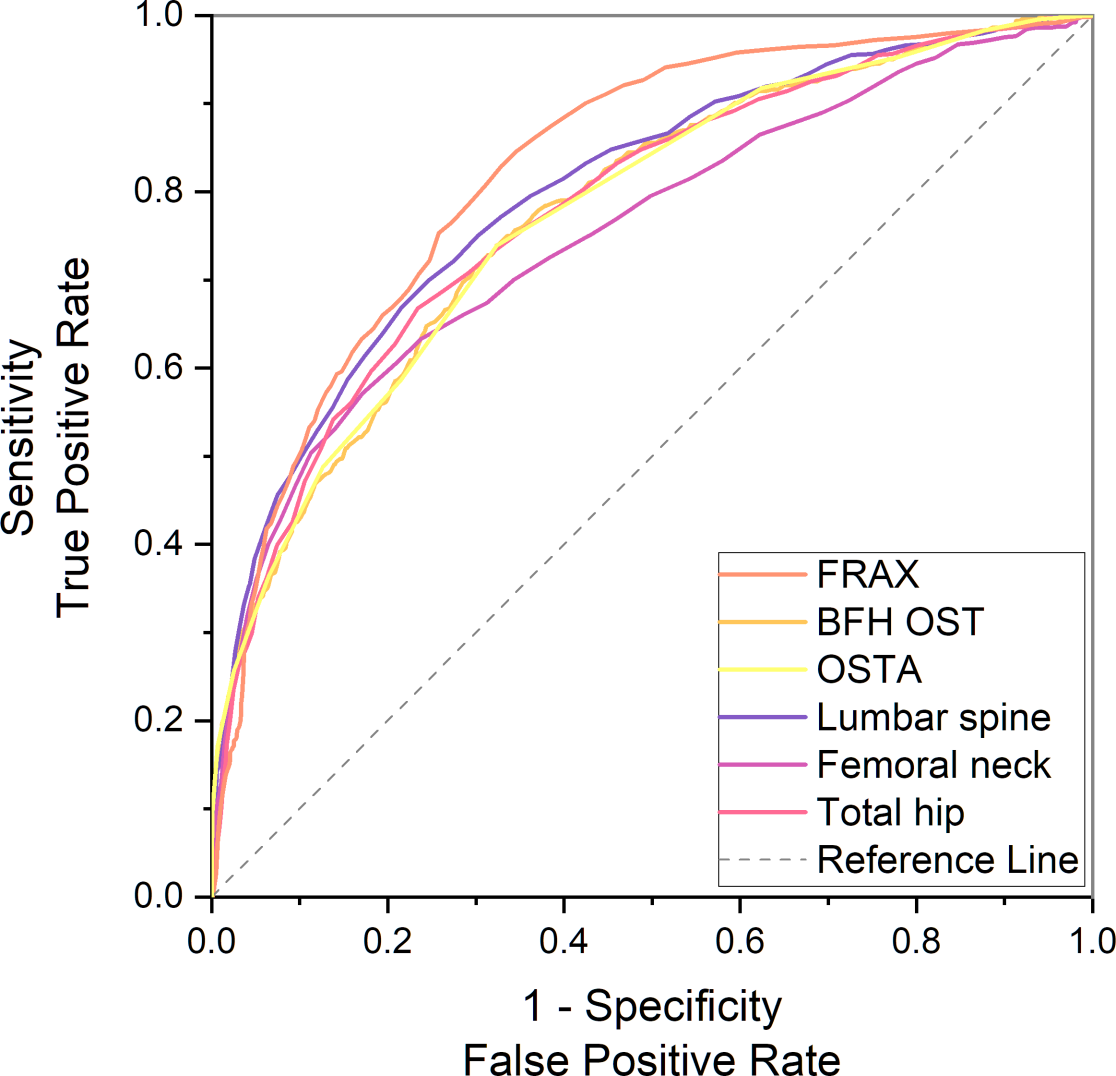
Comparison of different AUCs, including FRAX without BMD, BFH-OST, OSTA, and BMD T-score for identifying PNOVFs.

In the PNOVFs group, 58.6%, 53.2%, and 36.3% of women were found to have osteoporosis at the lumbar spine, femoral neck, and total hip, respectively (defined as BMD T-scores ≤ −2.5; **Figure 2**). The AUCs of BMD for identifying PNOVFs at the level of the total hip, femoral neck, and lumbar spine were 0.780, 0.753, and 0.799, respectively, with optimal cutoffs of −1.6, −2.4, and −2.2 (P<0.001 for all). The AUC of FRAX (without BMD) was 0.825, with an optimal cutoff of 3.6%. The AUC of the OSTA was 0.774, with an optimal cutoff of -1. The area under the curve of the BFH-OST was 0.775, with an optimal cutoff of 13.3 (**Table 4** and **Figure 3**). The comparison of the four tools is shown in **Figure 4**.

**Table 4:** AUC and sensitivity and specificity values of the FRAX, BMD T-score, OSTA, and BFH-OST for identifying PNOVFs.

Parameter	AUC (95% CI)	Z	P-value	Cutoff	Sensitivity, %	Specificity, %	+LR, %	−LR, %
FRAX	0.825 (0.810 - 0.839)	36.054	<0.001	>3.6	82.92	67.09	2.52	0.25
BMD T-score								
Total hip	0.780 (0.765 - 0.795)	26.599	<0.001	≤-1.6	66.77	76.64	2.86	0.43
Femoral neck	0.753 (0.737 - 0.769)	21.767	<0.001	≤-2.4	57.14	82.87	3.34	0.52
Lumbar spine	0.799 (0.784 - 0.814)	29.377	<0.001	≤-2.2	66.77	78.52	3.11	0.42
OSTA	0.774 (0.758 - 0.789)	26.253	<0.001	≤-1	73.91	67.62	2.28	0.39
BFH-OST	0.775 (0.760 - 0.791)	26.196	<0.001	≤13.3	73.91	67.67	2.29	0.39

BMD, bone mineral density; BFH-OST, Beijing Friendship Hospital Osteoporosis Screening Tool; OSTA, Osteoporosis Self-Assessment Tool for Asians;FRAX, Fracture Risk Assessment Tool; AUC, area under the receiver operating characteristic curve; PNOVFs, painful new osteoporotic vertebral fractures; CI, confidence interval; +LR: positive likelihood ratio; -LR: negative likelihood ratio.

## Discussion

This study retrospectively assessed and compared the performance of BMD, OSTA, FRAX (without BMD), and BFH-OST in identifying PNOVFs in postmenopausal Chinese populations. The mean height, weight, and BMI were lower in the PNOVFs group than in the control group, whereas the mean age, previous fracture, history of rheumatoid arthritis, and history of glucocorticoid use were higher in the PNOVFs group than in the control group. This finding is consistent with the results of previous studies (6, 15, 16). Conversely, no significant difference was found in parent hip fracture and alcohol consumption between the PNOVF and control groups. The lower height in the PNOVFs group may be attributed to the height loss of the vertebra or kyphotic deformity caused by vertebral compression fracture.

BMD measured using dual-energy DXA is the gold standard for diagnosing osteoporosis. Furthermore, it has been reported that BMD is an important determinant of bone strength, and its value could represent approximately 70% of bone strength. Osteoporosis can be diagnosed when the BMD T-scores are ≤-2.5. In this study, the prevalence of osteoporosis ranged from 36.3% to 72.3%, according to different criteria in PNOVFs population. The prevalence was 72.3% at the any site, 58.6% at the lumbar spine site, 53.2% at the femoral neck site and 36.3% at the total hip sites. The T-scores of the femoral neck, total hip, and lumbar spine in the PNOVFs group were significantly lower than those in the control group (all P < 0.001). When BMD was applied to identify PNOVFs, the AUC of femoral neck BMD, total hip BMD, and lumbar spine BMD were 0.753, 0.780, and 0.799, respectively with corresponding optimal cutoff values of −2.4, −1.6, and −2.2, sensitivities of 57.14%, 66.77%, and 66.77%, and specificities of 82.87%, 76.64%, and 78.52%. The specificity of BMD measurement in identifying PNOVFs was high, but its sensitivity was low; thus, it cannot be used as a screening tool for PNOVFs. Furthermore, BMD measurement requires dual-energy X-ray equipment, which is not feasible for community and primary medical institutions.

In this study, FRAX showed the best identification performance. The AUC of FRAX in screening PNOVFs was 0.825, with an optimal cutoff of 3.6%, a sensitivity of 82.92%, and a specificity of 67.09%. The efficacy and sensitivity of FRAX were preferable at a cut-off value of 3.6%. However, the National Osteoporosis Foundation (NOF) recommends that pharmacologic treatments should be initiated when an individual’s 10-year hip fracture probability is ≥3%, or 10-year major osteoporosis-related fracture probability ≥20% (10). As such, the optimal threshold in this study was much lower than the NOF threshold. Liu et al. previously found that the sensitivity and specificity of the FRAX with a threshold of 4.95% were 0.76 and 0.69, respectively, which is similar to the results of our study (11). Crandall et al. found that the performance of FRAX is unsatisfactory based on dichotomous cut-offs, and threshold-based approaches should be reassessed, particularly in younger women (12). Thus, threshold adjustments are required before the application of the FRAX tool in Chinese local practice. In addition, the calculation of FRAX scores requires relevant software and a certain amount of clinical data; thus, it is not convenient for community and primary medical institutions to screen for PNOVFs.

The OSTA was developed by Koh to identify osteoporosis in Asian women based on age and body weight (14). The distribution of OSTA scores between women with PNOVFs and the control group was significantly different. The discriminating ability of OSTA for identifying PNOVFs was found to be moderately predictive (AUC=0.774) at the optimal cutoff of -1, with an acceptable sensitivity of 73.91% and a specificity of 67.62%. This finding is consistent with the results of a previous study. Although the identifying value of OSTA is not as good as that of FRAX, its calculation includes only two clinical risk factors and this tool is more convenient for application in communities and primary medical institutions.

BFH-OST is an osteoporosis screening tool developed by the Beijing Friendship Hospital. The calculation of the BFH-OST includes the following four clinical risk factors: body height, weight, age, and previous fracture. The efficacy, sensitivity, and specificity of the BFH-OST for identifying osteoporosis have all been validated in previous studies. However, the ability of BFH-OST to screen for PNOVFs remains unclear. In this study, the AUC of BFH-OST in screening for PNOVFs was 0.775, with an optimal cutoff of 13.3, a sensitivity of 73.91, and a specificity of 67.67. The sensitivity of the BFH-OST is higher than that of the BMD of the total hip, femoral neck, and lumbar spine. BFH-OST could not only identify osteoporosis, but also PNOVFs in postmenopausal women. Although the sensitivity of BFH-OST is lower than that of FRAX in identifying PNOVFs, it has the advantage of simple calculations, and is suitable for communities and primary medical institutions to screen PNOVFs.

This study has several advantages. First, this study provides the first comparison of the four tools for identifying PNOVFs in postmenopausal women. Second, strict inclusion and exclusion criteria were introduced to rule out possible selection bias. In addition, we chose a community-enrolled population as the control group, which may be helpful for community screening. Furthermore, the selection of the clinical population was Han Chinese; thus, the calculated thresholds may not be applicable in other populations.

This study has some limitations. First, it had a retrospective design, and therefore future prospective studies are warranted to validate the results. Moreover, this was a single-center study and only included postmenopausal women in Beijing, and thus it cannot represent the overall population characteristics of China. Future multicenter, multi-regional, and multi-ethnic sample studies are therefore essential.

## Conclusion

Overall, the results of this study indicate that BMD is not sufficiently effective to identify PNOVFs in clinical practice. FRAX may be a preferable tool for identifying PNOVFs in postmenopausal women. Furthermore, BFH-OST and OSTA may be simple screening tools for PNOVFs.

## Data availability statement

The raw data supporting the conclusions of this article will be made available by the authors, without undue reservation.

## Ethics statement

The studies involving human participants were reviewed and approved by Ethics Committee of Beijing Friendship Hospital, Capital Medical University. The patients/participants provided their written informed consent to participate in this study.

## Author contributions

Each author made substantial contributions to this work. SJG, NA and QF contributed to the conception and design of the work. SJG and NA contributed to the acquisition of study data. SJG, NA, JSL contributed to the analysis and interpretation of data. ZHF, YY, and HM revised this article. All authors have drafted the work or substantively revised it. All authors contributed to the article and approved the submitted version.

## Conflict of interest

The authors declare that the research was conducted in the absence of any commercial or financial relationships that could be construed as a potential conflict of interest.

## Publisher’s note

All claims expressed in this article are solely those of the authors and do not necessarily represent those of their affiliated organizations, or those of the publisher, the editors and the reviewers. Any product that may be evaluated in this article, or claim that may be made by its manufacturer, is not guaranteed or endorsed by the publisher.
